# Synthesis and thermoelectric properties of Re_3_As_6.6_In_0.4_ with Ir_3_Ge_7_ crystal structure

**DOI:** 10.3762/bjnano.4.52

**Published:** 2013-07-17

**Authors:** Valeriy Y Verchenko, Anton S Vasiliev, Alexander A Tsirlin, Vladimir A Kulbachinskii, Vladimir G Kytin, Andrei V Shevelkov

**Affiliations:** 1 Department of Chemistry, Lomonosov Moscow State University, Moscow 119991, Russia; 2 National Institute of Chemical Physics and Biophysics, 12618 Tallinn, Estonia; 3 Faculty of Physics, Lomonosov Moscow State University, Moscow 119991, Russia

**Keywords:** band-structure calculations, energy conversion, Ir_3_Ge_7_ type, solid solution, thermoelectric material

## Abstract

The Re_3_As_7−_*_x_*In*_x_* solid solution was prepared for *x* ≤ 0.5 by heating the elements in stoichiometric ratios in evacuated silica tubes at 1073 K. It crystallizes with the Ir_3_Ge_7_ crystal structure, space group *Im*−3*m*, with a unit-cell parameter *a* ranging from 8.716 to 8.747 Å. The crystal structure and properties were investigated for a composition with *x* = 0.4. It is shown that indium substitutes arsenic exclusively at one crystallographic site, such that the As–As dumbbells with *d*_As–As_ = 2.54 Å remain intact. Re_3_As_6.6_In_0.4_ behaves as a bad metal or heavily doped semiconductor, with electrons being the dominant charge carriers. It possesses high values of Seebeck coefficient and low thermal conductivity, but relatively low electrical conductivity, which leads to rather low values of the thermoelectric figure of merit.

## Introduction

Thermoelectric materials with good efficiency are highly awaited by modern power engineering. Utilizing either the Seebeck or Peltier effects, it is possible to produce electricity from waste heat (e.g., that stemming from combustion in car engines) or to cool an environment under an external power supply. However, the efficiency of these processes depends on the efficiency of the thermoelectric material in question, which is defined by the value of the figure of merit 
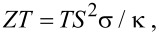
 where *T* is the absolute temperature, *S* the Seebeck coefficient, σ the electrical conductivity, and κ the thermal conductivity. It is shown in the literature [1] that the best thermoelectric materials are to be sought among narrow-gap semiconductors composed of heavy elements, in which structural features favor low thermal conductivity [2]. Attempts to improve the *ZT* value have led to the investigation of various types of thermoelectrics beyond the long-known lead and bismuth tellurides [3–4]. Among new candidates are the filled skutterudites [5–6], semiconducting clathrates [7], disordered materials such as Zn_4_Sb_3_ [8], and various inorganic and intermetallic compounds with complex crystal structures [9–10]. Compounds with the Ir_3_Ge_7_ structure type, namely Mo_3_Sb_5+δ_Te_2−δ_ [11], Nb_3_Sb_2_Te_5_ [12] and Re_3_As_7−_*_x_*Ge*_x_* [13], belong to the latter type of potential thermoelectric materials and have recently shown promising *ZT* values.

All members of the Ir_3_Ge_7_ family crystallize in the cubic space group *Im*−3*m* and feature the M–M dumbbells inside the Archimedean antiprism of the non-transition-element atoms. The strong hybridization of the transition-metal d-orbitals with the p-orbitals of a non-transition element may lead to the opening of a band gap near the Fermi level [14]. As a result, compounds with 55 valence electrons per formula unit (f.u.) exhibit semiconductor-like behavior. The number of valence electrons can be tuned through the solid-solution formation. For instance, Mo_3_Sb_5_Te_2_ and Re_3_As_6_Ge adopt 55 *e*^−^ per f.u. and should be semiconductors according to the band structure calculations. Their doped analogues, Mo_3_Sb_5.4_Te_1.6_ and Re_3_As_6.4_Ge_0.6_, display minor deviation from 55 *e*^−^ per formula. They behave as heavily doped semiconductors and possess the *ZT* values of 0.8 at 1050 K and 0.3 at 700 K, respectively [13]. To expand the Ir_3_Ge_7_ family and search for new promising thermoelectrics, we investigated different ways of obtaining new solid solutions based on Re_3_As_7_ or Mo_3_Sb_7_ compounds. In this paper, we report on the synthesis of the Re_3_As_7−_*_x_*In*_x_* solid solution (*x* ≤ 0.5), its crystal and electronic structures, and its thermoelectric properties.

## Experimental

### Synthesis and analysis

Rhenium (–325 mesh, 99.99%, Alfa Aesar) and arsenic (–70 mesh, 99.99%, Alfa Aesar) powders and indium ingots (99.95%, Sigma Aldrich) were used as received. Phase purity of the starting materials was checked by using the standard X-ray diffraction technique, and in all cases no impurity phases were found. To synthesize the title solid solution, stoichiometric quantities of the starting elements were heated in evacuated silica tubes at 1073 K for 7 days with further cooling to room temperature in a shut off furnace. Firstly, the samples were analyzed by means of X-ray powder diffraction using a Stoe STADI-IP diffractometer with Cu Kα_1_ radiation (Ge monochromator, λ = 1.540598 Å). To evaluate the lattice constants of the Re_3_As_7−_*_x_*In*_x_* solid solution, all X-ray diffraction patterns were recorded with Ge as an internal standard (*a* = 5.6576 Å). The data were treated with the program package Stoe WinXPOW. Secondly, the obtained samples were analyzed with a JSM JEOL scanning electron microscope operated at 20 kV and equipped with an EDX detection system INCA x-Sight. Both point-spectra acquisition and element mapping were used to investigate the elemental and phase composition of the samples.

### Structure determination

The crystal structure was determined by the Rietveld method from the X-ray powder diffraction data. For the sample with the nominal composition Re_3_As_6.6_In_0.4_, hereafter sample **S1**, the data were recorded with the Bruker D8 Advance diffractometer, Cu Kα_1_ radiation (Ge monochromator, λ = 1.540598 Å). For the Rietveld refinements we used the TOPAS software (version 4.2, Bruker-AXS). The refinement enabled us to determine minor quantities of three impurity phases (Table 1) that were taken into account during the subsequent refinement. The atomic parameters taken from the crystal structure of Re_3_As_7_ [15] were used as the starting model. The refinement showed that the unique position of the rhenium atom was fully occupied. One of the two positions of the arsenic atoms, namely, the 12d site, showed a remarkably low atomic displacement parameter and was subsequently refined as jointly occupied by indium and arsenic. The refinement led to the composition Re_3_As_6.70(3)_In_0.30(3)_ in reasonable agreement with the starting (synthetic) composition. Crystallographic details of the refinement are shown in Table 1, and the atomic parameters are shown in Table 2. Selected interatomic distances are listed in Table 3.

**Table 1:** Crystallographic data from the powder diffraction experiment for **S1**.

refined composition	Re_3_As_6.70(3)_In_0.30(3)_

formula weight (g·mol^−1^)	1095.041
*T* (K)	300
wavelength (Å)	1.540598
space group	*Im*−3*m* (No. 229)
cell dimensions, *a* (Å)	8.74231(6)
*V* (Å^3^)	668.157(14)
no. of formula units per cell	4
calculated density (g·cm^−3^)	10.88
2θ range (°)	17.00–85.01
*R*_p_, *R*_wp_, GOF	0.056, 0.077, 1.4
impurity phases (weight %)	Re 2.0%, InAs 2.3%, In_2_O_3_ 1.0%

**Table 2:** Atomic coordinates and displacement parameters for **S1**.

site	Wyck.	*x*	*y*	*z*	*B*_iso_ (Å^2^)	occupancy

Re	12e	0.3396(2)	0	0	0.60(3)	1Re
E1	12d	1/4	0	1/2	0.82(11)	0.90(1)As + 0.10(1)In
As2	16f	0.1662(2)	0.1662(2)	0.1662(2)	0.82(5)	1As

**Table 3:** Selected interatomic distances for **S1**.

bond	distance (Å)

Re–E1 × 4	2.597(1)
Re–As2 × 4	2.553(1)
Re–Re × 1	2.805(3)
As2–As2 × 1	2.539(5)
As2–As2 × 3	2.905(3)

### Electronic-structure calculations

The FPLO (full potential local orbitals) code was utilized for the electronic-structure calculations [16]. FPLO performs density functional calculations with the local density approximation (LDA) for the exchange–correlation potential [17]. The crystallographic data presented in Table 4 were used for the calculations [15]. The integrations in the *k* space were performed by an improved tetrahedron method [18] on a grid of 16 × 16 × 16 *k* points evenly spread in the first Brillouin zone.

**Table 4:** Re_3_As_7_ crystallographic data used for electronic-structure calculations [15].

Space group *Im*−3*m* (No. 229), *a* = 8.7162(7) Å				

site	Wyck.	*x*	*y*	*z*

Re	12e	0.3406(9)	0	0
As1	12d	1/4	0	1/2
As2	16f	0.1687(20)	0.1687(20)	0.1687(20)

### Physical property measurements

For thermal transport measurements, the sample **S1** was thoroughly ground and pressed at room temperature into a rectangular pellet of dimensions 8 × 3 × 2 mm^3^. The density of **S1** was estimated from the linear sizes of the pellet to be about 70% of the theoretical density. This pellet was used to measure the electrical conductivity (σ), the Seebeck coefficient (*S*), and the thermal conductivity (κ) in the temperature range of 77–300 K in a home-built setup. Resistance was determined from the voltage drops by applying a four-probe method in accordance with Ohm’s law, i.e., *R =* Δ*V*/*I*. The current (*I*) was scanned in the range between 2.5 µA and 16 mA, and subsequently σ was calculated after measuring the length between the contacts (*L*) according to σ *= L/(AR)*, with the area *A* = 3 × 2 mm^2^. The Seebeck coefficient and thermal conductivity were measured by using an internal standard to determine the temperature difference in a custom-designed sample puck that was plugged into the cold finger of a closed-cycle refrigerator. All measurements were performed under dynamic vacuum.

For the magnetization measurements, powder samples of Re_3_As_7_ and **S1** were loaded into plastic capsules. Measurements were performed with the VSM setup of Quantum Design PPMS in external fields of 0.1, 0.5, 1, 2, and 5 T. To estimate the diamagnetic contribution from the sample holder, an empty capsule was measured under the same conditions.

## Results and Discussion

### Synthesis, sample characterization and crystal structure

**Figure 1:**
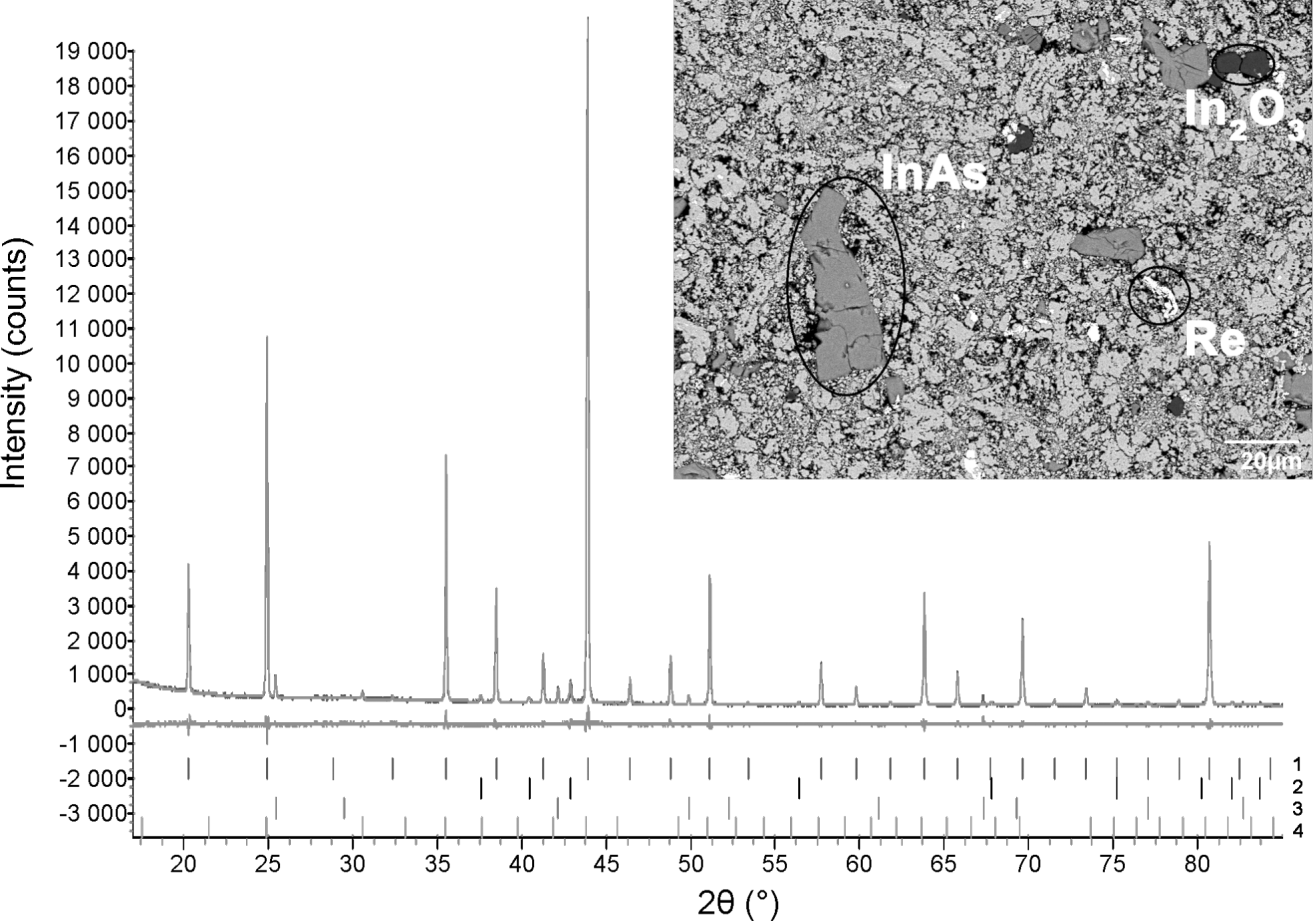
The plot of Rietveld refinement for the **S1** sample. Experimental and difference curves, and positions of Bragg peaks are shown on the plot. Marked with numbers: 1: Re_3_As_6.70(3)_In_0.30(3)_; 2: Re; 3: InAs; 4: In_2_O_3_. Inset: SEM micrograph of **S1** showing the distribution of secondary phases in the microstructure (the most contaminated portion was chosen for showing all three admixtures).

**Figure 2:**
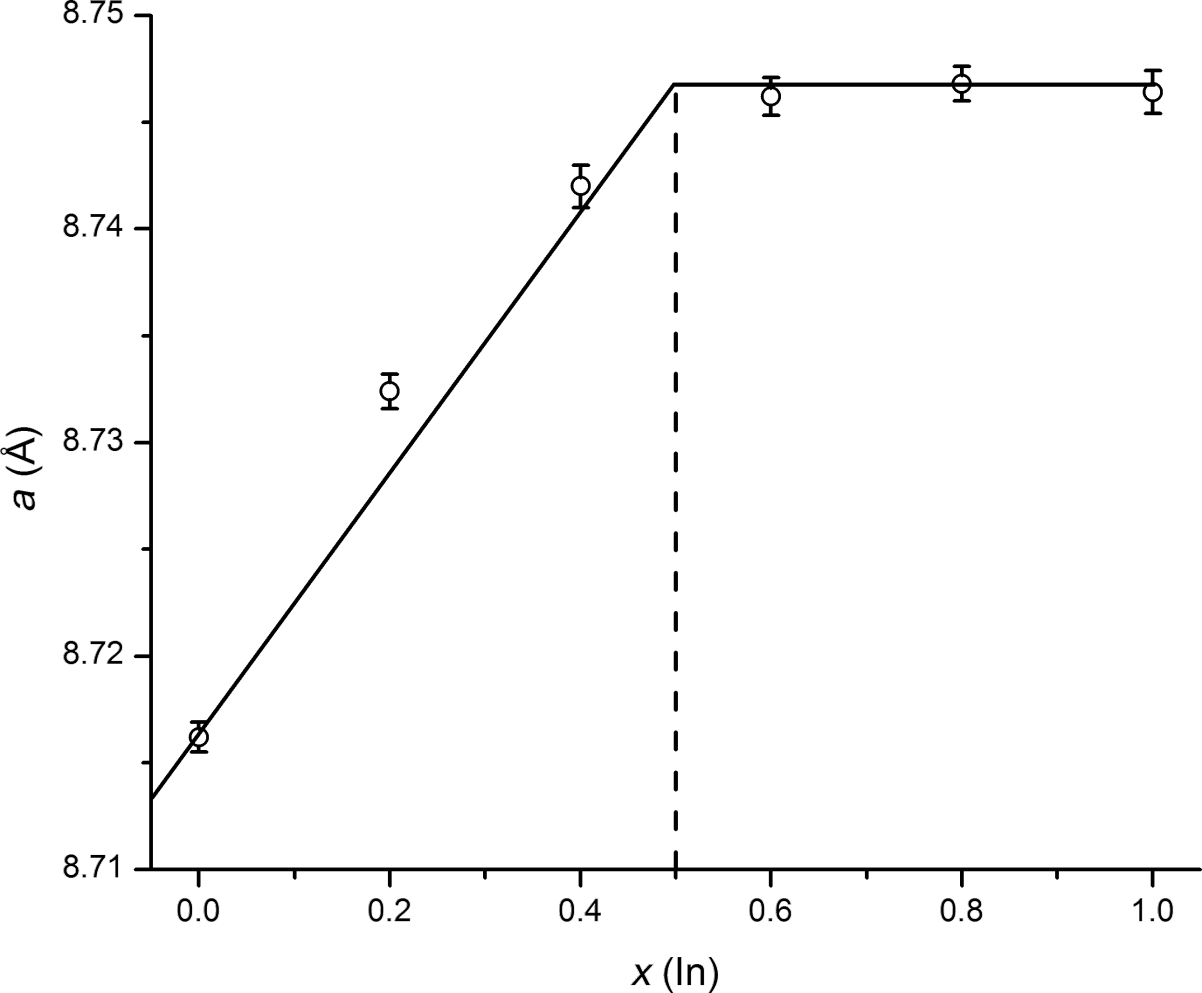
Dependence of the Re_3_As_7−_*_x_*In*_x_* cubic-unit-cell parameter on the nominal indium content. Esd’s are calculated from least-squares fits of the powder data.

The synthesis of the Re_3_As_7−_*_x_*In*_x_* series with *x* = 0, 0.2, 0.4, 0.6, 0.8, and 1 from pure elements resulted in black powders that were stable in air. The obtained samples were analyzed by X-ray powder diffraction. All samples showed reflections of the main phase of the Re_3_As_7_ type (space group *Im*−3*m*), together with minor reflections of Re, InAs, and In_2_O_3_ admixtures, the presence of which was also confirmed with EPMA (Figure 1). In order to obtain single-phase samples, we tried to improve the synthetic procedure, but neither increasing the annealing time nor pressing the reactants into pellets led to phase-pure samples. Some general trends should be noted. For the samples with 0 ≤ *x* ≤ 0.4, absolute intensities and, thus, quantities of admixtures remain constant, while for *x* > 0.5, quantities of Re and InAs start to increase. Additionally, we found by a linear interpolation that the unit cell parameter of the Re_3_As_7−_*_x_*In*_x_* solid solution increases up to *x* = 0.5, and then remains constant at higher *x* (Figure 2). All these facts suggest that the solid solution in question exists only for *x* ≤ 0.5. The outermost composition Re_3_As_6.5_In_0.5_ possesses exactly 55 valence electrons per formula unit. As mentioned above, this electron concentration should yield the semiconducting behavior for compounds with the Ir_3_Ge_7_ structure type. Thus, the indium substitution for arsenic in Re_3_As_7_ could be used as a chemical modification to control transport properties of this system.

**Figure 3:**
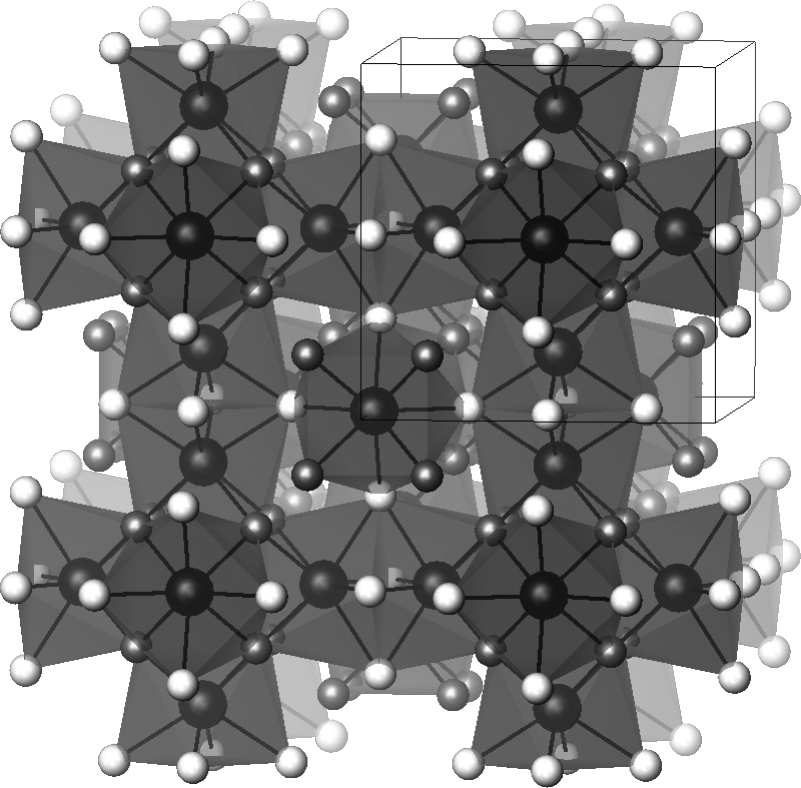
Polyhedral view of the Re_3_As_7−_*_x_*In*_x_* crystal structure. Re is shown as black spheres inside the polyhedra, E1: white spheres, and As2: gray spheres at the vertices.

The crystal structure of the solid solution was studied for the **S1** sample by the Rietveld method from X-ray powder diffraction data (Figure 1, Table 1 and Table 2). The title compound crystallizes with the Ir_3_Ge_7_ crystal structure (Figure 3). This structure can be described as being composed of rhenium-centered square antiprisms of E atoms, ReE_8_ (E = As/In). Two square antiprisms are linked by sharing a square face. These pairs form the so-called Re_2_E_12_ barrels, the main building blocks of the crystal structure. The barrels, oriented along the main crystallographic directions, form two interpenetrating 3D networks in accordance with the body-centering and, thus, build up the entire crystal structure.

**Figure 4:**
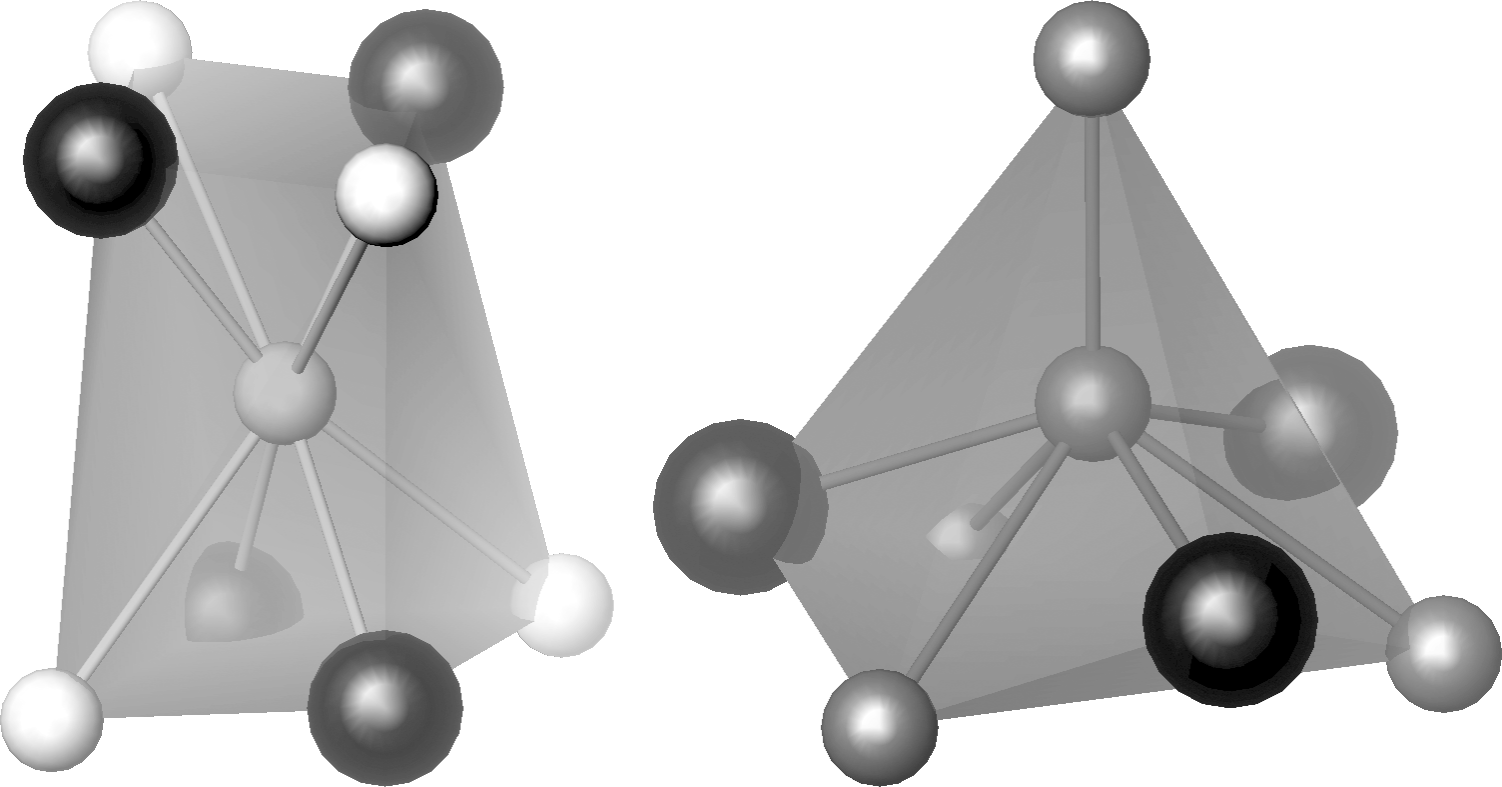
Coordination polyhedra of E1 (left) and As2 (right) sites in the crystal structure of Re_3_As_7−_*_x_*In*_x_*.

The formation of the solid solution may be associated with a chemical substitution on different crystallographic sites. There are two sites forming the coordination polyhedra of E atoms in the Ir_3_Ge_7_ structure type (Figure 4), and the substitution is possible for both sites depending on the chemical nature of the E elements. It is known from the literature that in the case of the Ge for As substitution in the parent compound Re_3_As_7_, all Ge atoms enter the As2 (16f) site [13]. In contrast, we have found that when indium substitutes for arsenic in Re_3_As_7_, all indium atoms are on the E1 (12d) site. The preference for the certain As position depends on different aspects, including size, nuclear charge, and number of valence electrons of the heteroatom. In particular, there is an E–E single bond between atoms occupying the 16f site, with a bond distance of 2.538(5) Å. Clearly, indium does not favor such a short bond to arsenic and, therefore, avoids the occupation of this site.

### Electronic structure, magnetic and thermoelectric properties

**Figure 5:**
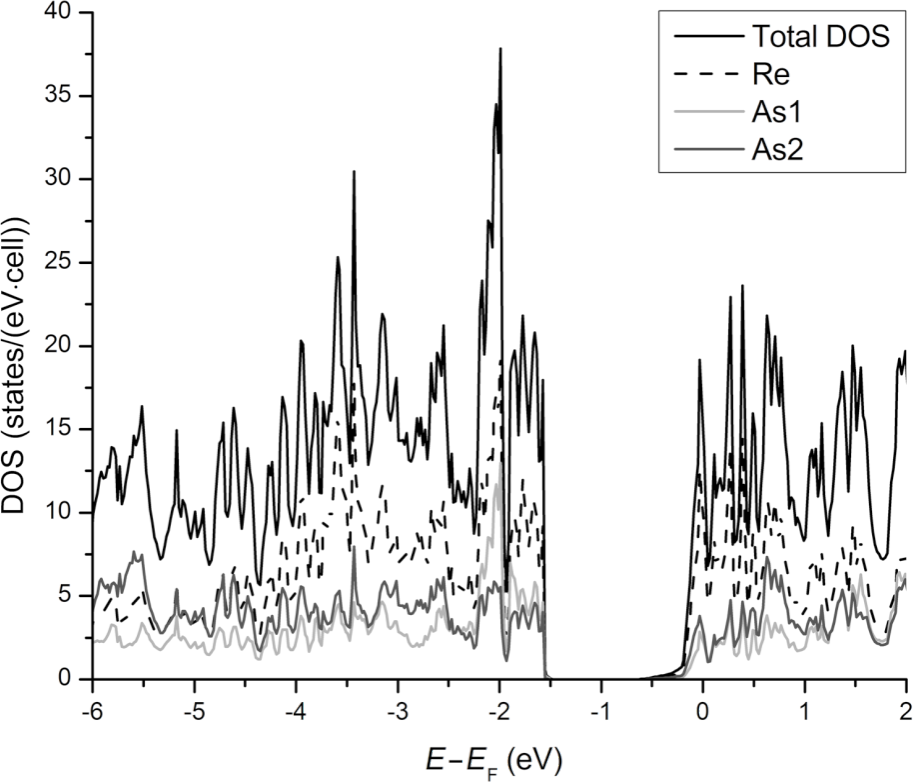
Density-of-states curve for Re_3_As_7_. Re contribution: dashed line, As1 and As2: light and dark gray lines, respectively.

The computed density of states for Re_3_As_7_ is shown in Figure 5. The Fermi level lies slightly above the gap of 0.8 eV that separates the conduction band from the valence band. The nonzero DOS at *E*_F_ implies metallic behavior for the undoped Re_3_As_7_. Additionally, the steep slope of the DOS curve near *E*_F_ should lead to a high Seebeck coefficient according to *S ~* 1/*N∙*∂*N(E*_F_*)/*∂*E* [19], provided that the system is made semiconducting by doping. Indeed, the absolute values of *S* for Re_3_As_6.4_Ge_0.6_ exceed 150 µV·K^−1^ at high temperatures, thus leading to high values of *ZT* [13].

In Re_3_As_7_, the calculated density of states at *E = E*_F_ is 8.3 states/(eV·f.u.). For the solid solution Re_3_As_6.7_In_0.3_ (the composition obtained from the Rietveld refinement of the X-ray powder diffraction data, see Table 1), the DOS is reduced to 5.15 states/(eV·f.u.), given the rigid-band shift with the assumption that Re_3_As_7_ possesses 56 valence electrons per f.u. and Re_3_As_6.7_In_0.3_ 55.4 electrons. Therefore, both compounds should be metallic with a Pauli paramagnetic contribution to the total susceptibility χ = χ_dia_ + χ_P_, where χ*_dia_* is core diamagnetism, and χ_P_ = μ_B_^2^·*N*(*E*_F_), with μ_B_ being the Bohr magneton [20]. The formula yields χ_P_ = 9 × 10^−5^ and 5.5 × 10^−5^ emu/mol for Re_3_As_7_ and **S1**, respectively.

**Figure 6:**
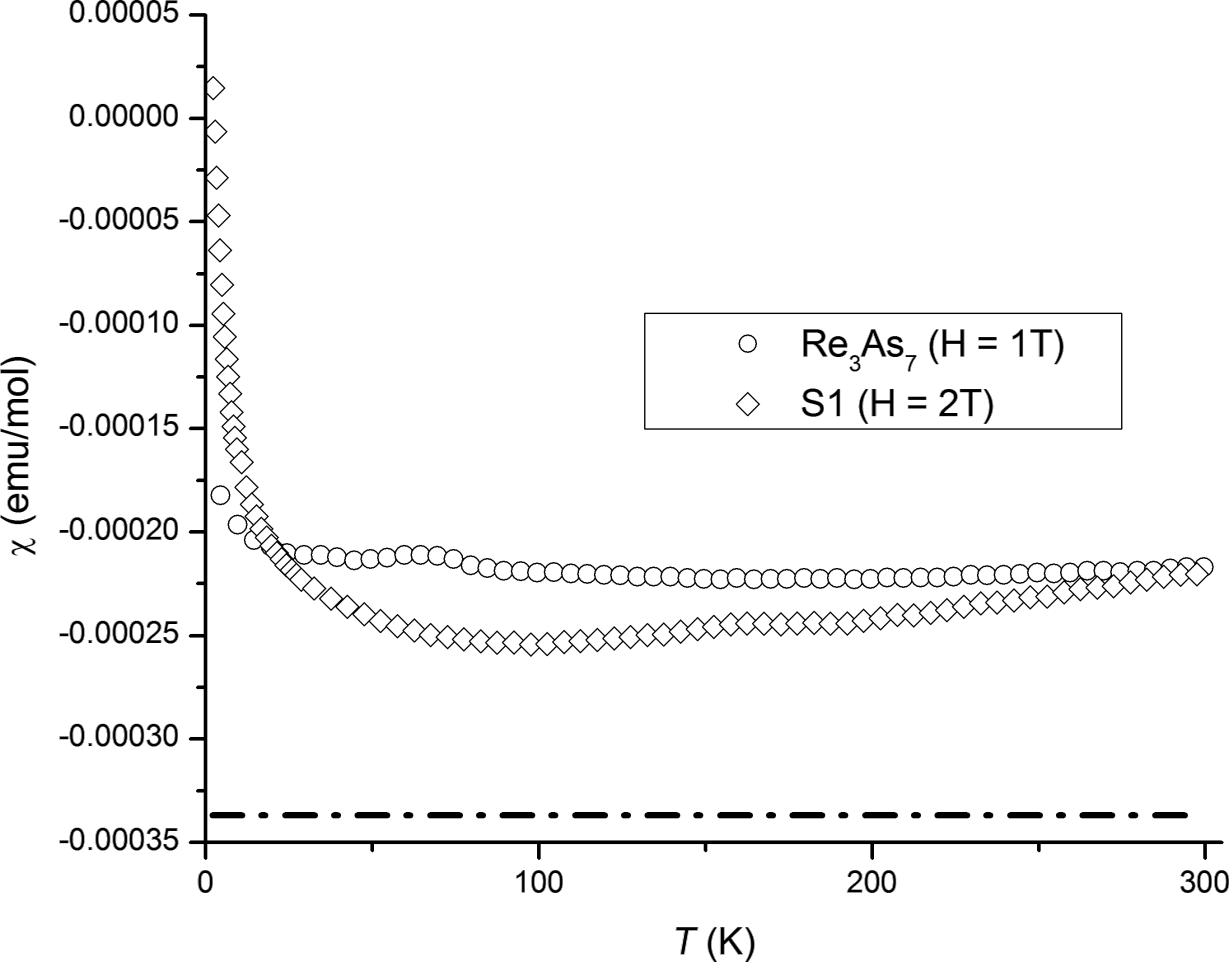
Magnetic susceptibility-versus-temperature plots for the Re_3_As_7_ and **S1** samples. The contribution of core diamagnetism is shown as a dash-dotted line.

Experimentally, both Re_3_As_7_ and **S1** show substantial diamagnetism in the examined temperature range. However, the susceptibility curves, Figure 6, lie above the level of core diamagnetism χ_dia_ = −3.37 × 10^−4^ emu/mol, computed for a combination of Re^7+^ and As(V) [21]. Therefore, both pure and In-doped Re_3_As_7_ feature an additional paramagnetic contribution to the susceptibility. The experimental value of χ_P_ = χ − χ_dia_ = 1.20(7) × 10^−4^ emu/mol for Re_3_As_7_ is reasonably close to the one expected from the DOS at *E*_F_. However, the calculation of χ_P_ substantially depends on the estimation method of χ_dia_, especially for a compound that cannot be considered as ionic. For this reason, the calculated value of χ_P_ may differ from the experimental one. The susceptibility of the **S1** slightly decreases upon cooling but starts increasing below 80 K. While the low-temperature upturn could be due to a small number of paramagnetic impurities, the conspicuous increase in χ above 80 K does not conform to the Pauli paramagnetism and reflects deviations of **S1** from a simple metal.

**Figure 7:**
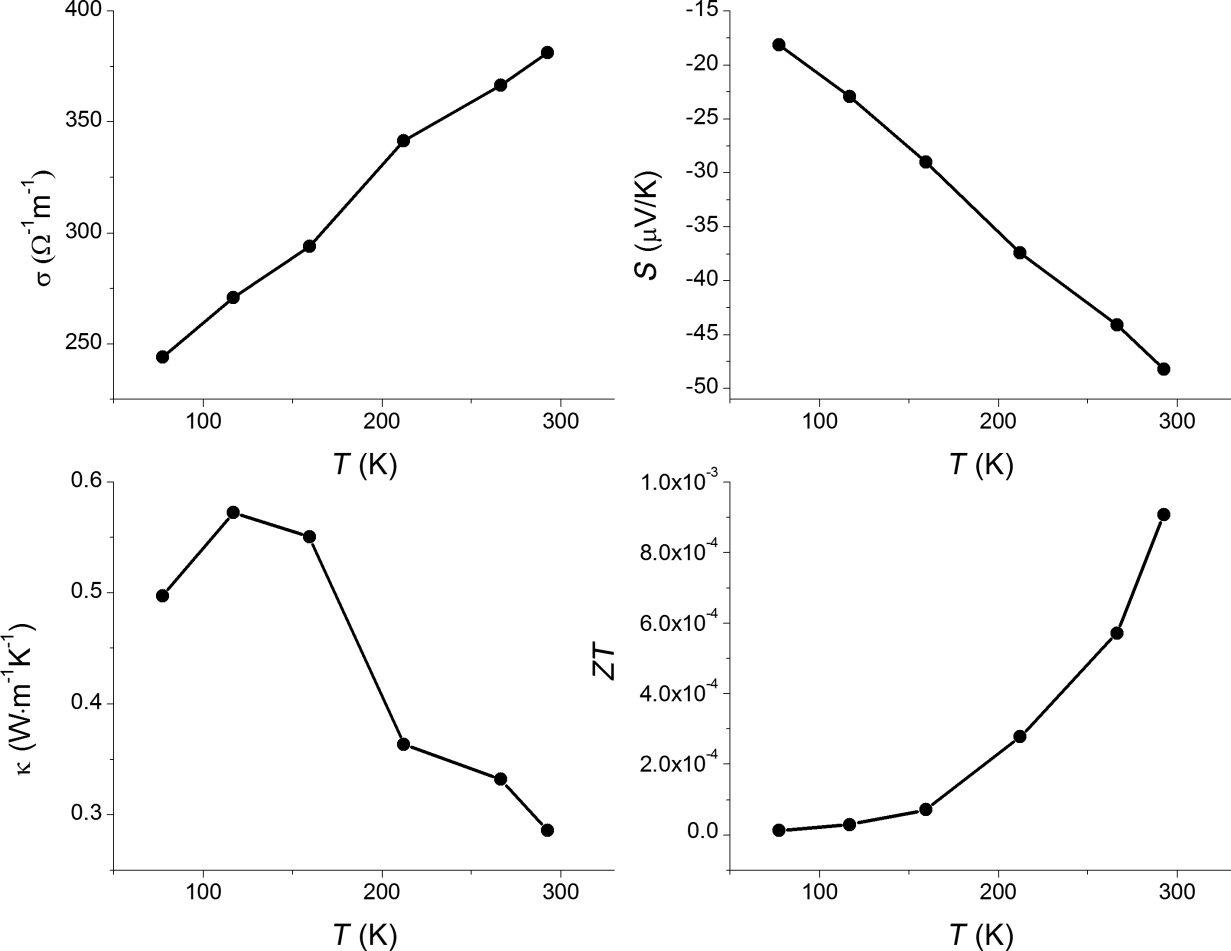
Thermoelectric properties of the **S1** sample as a function of temperature. Solid lines are drawn to guide the eye.

Figure 7 compiles the plots of the electric conductivity (σ), Seebeck coefficient (*S*), thermal conductivity (κ), and *ZT* in the temperature range of 77–300 K for **S1**. *S* is negative in this temperature range, evidencing that the **S1** is an n-type conductor. However, the σ-versus-*T* behavior for the **S1** sample is neither metallic nor classically semiconducting, because σ increases almost linearly with temperature. Thus, **S1** can be regarded as a bad metal or degenerate semiconductor, considering the possible presence of defects, such as vacancies in its crystal structure, which was proposed earlier for Re_3_As_7_ [15]. In the Ir_3_Ge_7_ family, Re_3_As_7−_*_x_*Ge*_x_* exhibits n-type conductivity [13], while Mo_3_Sb_5+δ_Te_2−δ_ is a p-type conductor [11]. The obtained values of *S* for **S1** are comparable with those for Re_3_As_6.4_Ge_0.6_ and Mo_3_Sb_5.4_Te_1.6_: −49, −72, and +55 µV·K^−1^ at 300 K, respectively [11,13]. Moreover, the extent of the substitution *x* in the Re_3_As_7−_*_x_*In*_x_* solid solution can be further optimized, and possibly lead to larger values of *S*. Unfortunately, the **S1** displays considerably lower values of the electrical conductivity compared to both Re_3_As_7−_*_x_*Ge*_x_* and Re_3_As_7−_*_x_*Sn*_x_* (0.1 ≤ *x* ≤ 0.6) [22]. For instance, the room-temperature value of 1090 Ω^−1^·cm^−1^ for Re_3_As_6.4_Ge_0.6_ [13] is about 3000 times larger than the observed value of 3.8 Ω^−1^·cm^−1^ for **S1**.

The thermal conductivity of the sample **S1** is quite low. Its room-temperature value is 0.3 W·m^−1^·K^−1^, which is an order of magnitude lower than for the Ge- and Sn-substituted compounds. This may be caused by two factors: Firstly, it could be attributed to the preference of indium atoms for only one position within the crystal structure (increased structural complexity); secondly, relatively low density of the sample (about 70%) may diminish the thermal conductivity due to the sample porosity. The total thermal conductivity is a sum of the electronic (κ_e_) and lattice (κ_L_) parts. Taking into account the rather low electrical conductivity and applying the Wiedemann–Franz relation κ_e_* =* σ*LT*, where *L* is the ideal Lorentz number, we estimate that the electronic part of the total thermal conductivity is negligibly small, and the observed value is essentially the lattice contribution to the thermal conductivity.

Combining the electrical conductivity, Seebeck coefficient and thermal conductivity, we calculate the temperature dependence of *ZT* shown in Figure 7. *ZT* increases with temperature, and reaches *ZT* = 0.0008 at room temperature, which is 30 times lower than for Re_3_As_7−_*_x_*Ge*_x_* [13]. Given the compositional width of the Re_3_As_7−_*_x_*In*_x_* solid solution and the low thermal conductivity of the investigated sample, we note that the optimum combination of *S* and σ for Re_3_As_7−_*_x_*In*_x_* is still to be found.

## Conclusion

Chemical modification of Re_3_As_7_ resulted in the formation of the new Re_3_As_7_-based solid solution Re_3_As_7−_*_x_*In*_x_* (*x* ≤ 0.5) with an Ir_3_Ge_7_ type of crystal structure. The indium for arsenic substitution occurs exclusively on the 12d site, thus keeping intact the As–As dumbbells with *d*_As–As_ = 2.538(5) Å. While Re_3_As_7_ shows a Pauli paramagnetic contribution to the magnetic susceptibility in line with the results of band-structure calculations, the **S1** sample behaves as a bad metal or heavily doped semiconductor, with electrons being the dominant charge carriers. This compound combines low thermal conductivity with a relatively low electrical conductivity, and therefore, its thermoelectric figure of merit *ZT* reaches only 0.0008 at room temperature. Further optimization of the thermoelectric properties by varying the chemical composition of Re_3_As_7−_*_x_*In*_x_* is proposed.
